# The CCR2/MCP-1 Chemokine Pathway and Lung Adenocarcinoma

**DOI:** 10.3390/cancers12123723

**Published:** 2020-12-11

**Authors:** Payal Mittal, Liqing Wang, Tatiana Akimova, Craig A. Leach, Jose C. Clemente, Matthew R. Sender, Yao Chen, Brandon J. Turunen, Wayne W. Hancock

**Affiliations:** 1 Chemical Biology, Medicinal Science Technology, GlaxoSmithKline, Collegeville, PA 19426, USA; pmittal9@its.jnj.com (P.M.); craig.a.leach@gsk.com (C.A.L.); JClemen4@ITS.JNJ.com (J.C.C.); matthew.r.sender@gsk.com (M.R.S.); yao.x.chen@gsk.com (Y.C.); brandon.j.turunen@gsk.com (B.J.T.); 2 Division of Transplantation Immunology, Department of Pathology and Laboratory Medicine, Children’s Hospital of Philadelphia, and Perelman School of Medicine, University of Pennsylvania, Philadelphia, PA 19104, USA; wangl@email.chop.edu (L.W.); Tatiana.Akimova@pennmedicine.upenn.edu (T.A.)

**Keywords:** MDSC, CCR2, lung cancer, anti-tumor immunity, immunotherapy

## Abstract

**Simple Summary:**

Myeloid-derived suppressor cells (MDSCs) are present at the sites of many solid tumors and dampen host immune responses. We studied how these MDSCs accumulate and how this process might be interrupted using lung cancer cell lines growing in immunocompetent mice. We found that tumor cells release a chemokine, MCP-1, that attracts MDSCs as a result of their expression of the MCP-1 receptor, CCR2. We showed that mice lacking CCR2, or the use of a small-molecule inhibitor of CCR2, prevented MDSC recruitment to the tumors and derepressed host T cell responses, allowing immune activation and inhibition of tumor growth. These data suggest that CCR2 inhibitors may be a new tool for cancer immunotherapy.

**Abstract:**

Host anti-tumor immunity can be hindered by various mechanisms present within the tumor microenvironment, including the actions of myeloid-derived suppressor cells (MDSCs). We investigated the role of the CCR2/MCP-1 pathway in MDSC-associated tumor progression in murine lung cancer models. Phenotypic profiling revealed maximal expression of CCR2 by tumor-resident MDSCs, and MCP-1 by transplanted TC1 tumor cells, respectively. Use of CCR2-knockout (CCR2-KO) mice showed dependence of tumor growth on CCR2 signaling. Tumors in CCR2-KO mice had fewer CCR2^low^ MDSCs, CD4 T cells and Tregs than WT mice, and increased infiltration by CD8 T cells producing IFN-γ and granzyme-B. Effects were MDSC specific, since WT and CCR2-KO conventional T (Tcon) cells had comparable proliferation and production of inflammatory cytokines, and suppressive functions of WT and CCR2-KO Foxp3+ Treg cells were also similar. We used a thioglycolate-induced peritonitis model to demonstrate a role for CCR2/MCP-1 in trafficking of CCR2+ cells to an inflammatory site, and showed the ability of a CCR2 antagonist to inhibit such trafficking. Use of this CCR2 antagonist promoted anti-tumor immunity and limited tumor growth. In summary, tumor cells are the prime source of MCP-1 that promotes MDSC recruitment, and our genetic and pharmacologic data demonstrate that CCR2 targeting may be an important component of cancer immunotherapy.

## 1. Introduction

T cells are the major drivers of anti-tumor immune responses but their functions within the tumor microenvironment can be impaired by immune cells with inhibitory properties, as well as through the actions of fibroblasts and endothelial cells [1]. One important immunosuppressive mechanism involves the actions of myeloid-derived suppressor cells (MDSCs) in the tumor microenvironment [2]. Once recruited, various cytokines, including vascular endothelial growth factor (VEGF) [3], granulocyte colony stimulating factor (G-CSF) and granulocyte/monocyte colony stimulating factor (GM-CSF) [4], are implicated in the expansion and maintenance of MDSCs within the tumor microenvironment.

MDSCs blunt anti-tumor immunity through multiple, sometimes surprising mechanisms. For example, they can directly contribute to tumor growth and vascularization by producing MMP9 and differentiating into endothelial cells [5]. They can also induce cross-talk between STAT3 and NOTCH signaling in cancer cells to confer properties of stemness [6]. Additionally, MDSCs can exert direct suppressive effects on various immune cells, including T cells, through their production of arginase (Arg1) and Arg1+ exosomes, nitric-oxide (NO) synthase enzymes, IL-10, TGF-β and indolamine 2,3-dioxygenase (IDO) [7,8]. They can also restrict CD8 T cell activity following antigenic stimulation by secreting reactive oxygen species [9,10]. In addition to suppressing T cell functions, MDSCs facilitate tumor progression through cell–cell contact with macrophages and inhibiting their IL-12 secretion [11], inhibiting NK cell-mediated cytotoxicity against tumors [12], and recruiting and inducing Foxp3+ T-regulatory (Treg) within the tumor microenvironment [13,14].

MDSCs are generated from common myeloid progenitor cells in bone marrow and are found abundantly in murine tumors [15] and human cancers such as glioblastoma [16], pancreatic adenocarcinoma [17] and breast cancer [18]. GM-CSF, G-CSF and IL-6 cytokines induce rapid generation of MDSCs in both murine and human bone marrow [19]. Tumor production of GM-CSF plays a crucial role in generation of MDSCs in mice [20] and in humans [21] by acting upon CD33+ peripheral blood mononuclear cells. The chemokine MCP-1 (CCL2) is important for the recruitment of bone marrow generated myeloid cells into the blood, and for the trafficking of MDSCs to tumor sites, by binding to the G protein-coupled receptor, CCR2 [22,23,24,25]. These observations provided a rationale for us to further assess the effects of MCP-1/CCR2 targeting on MDSC recruitment to tumors and concomitant effects on anti-tumor immunity and tumor growth.

## 2. Results

### 2.1. Baseline Studies of Myeloid and T Cell Lineages in CCR2RKO Mice

We planned to investigate the role of the CCR2/MCP-1 pathway in MDSC-associated tumor progression in murine syngeneic lung cancer models but undertook some basic characterization of the main mouse strain to be used, namely mice lacking the gene for CCR2 (CCR2KO). We found that CCR2 deletion did not affect thymic T cell development (Figure S1) or the frequencies of peripheral CD4+ and CD8+ T cells, including CD4+Foxp3+ T-regulatory (Treg) cells (Figure S2). Among myeloid cells, the frequency of splenic CD11b+Ly6C^high^ inflammatory monocytes was significantly compromised in CCR2KO vs. WT mice, whereas neutrophil populations were comparable (Figure S3). This led us to consider a developmental defect in the production of CD11b+Ly6C^high^ inflammatory monocytes but we found their frequency in bone marrow was comparable to WT mice (Figure S4). Hence, compared to WT mice, CCR2RKO mice appeared to have normal T cell populations but a marked decrease in the peripheral levels of CD11b+Ly6C^high^ monocytes. Previous studies have shown that MCP-1/CCR2 stimulation causes desensitization of the CXCL12–CXCR4 signaling that normally promotes bone marrow cell sequestration [26]. These findings are consistent with a requirement for CCR2 expression for egress of CD11b+Ly6C^high^ monocytes from the bone marrow [27].

### 2.2. Expression of CCR2 and CCL2 at the Tumor Site

**Figure 1:**
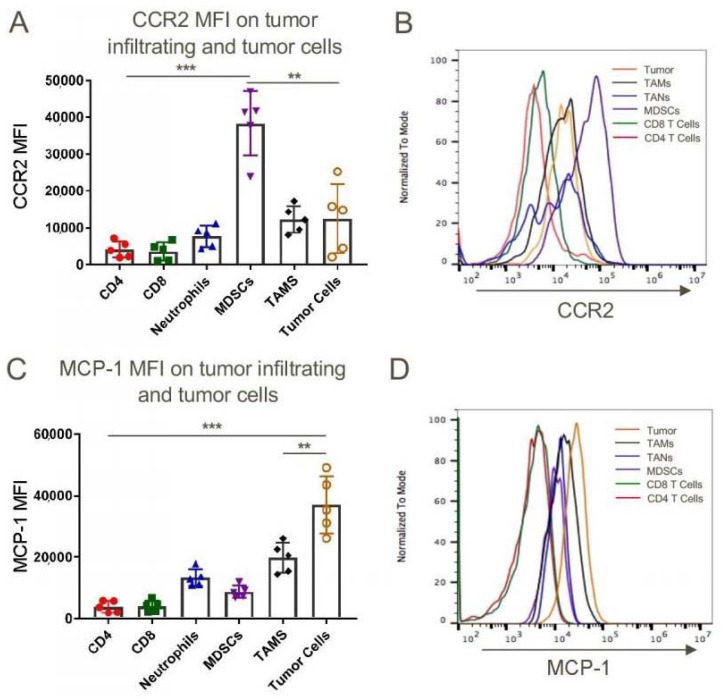
Myeloid-derived suppressor cells (MDSCs) derived from subcutaneously transplanted mouse lung epithelial cell tumor TC1 expressed the highest levels of chemokine CCR2 whereas tumor cells are the major source of CCL2 (MCP-1). WT C57BL/6 mice were injected subcutaneously with 1 million TC1 cells and tumors were harvested 21 days post-injection and were analyzed for CCR2 (**A**,**B**) and CCL2 (**C**,**D**) expression by flow cytometry; ** *p* < 0.01 and *** *p* < 0.001, *n* = 5/group. Data are representative of three independent experiments.

We began our tumor studies by assessing the expression of CCR2 and MCP-1/CCL2 within tumors that developed in syngeneic C57BL/6 mice after subcutaneous injection of a murine lung adenocarcinoma cell line, TC1. While there are differences in the flow cytometric phenotypic markers of relevant tumor-associated cell types between humans and mice, and even within murine studies, in the current work we defined MDSCs as CD11b+Ly6C^high^Ly6G^low^ cells, tumor-associated neutrophils (TAN) as CD11b+ Ly6C^intermediate^Ly6G^high^ cells, and tumor-associated macrophages (TAM) as CD11b+Ly6C^low^Ly6G^low^ cells. Using these definitions and the gating strategy outlined in Figure S5, we found that MDSCs expressed significantly more CCR2 than tumor-associated CD4 or CD8 T cells, TAN, TAM or tumor cells (Figure 1A,B), whereas the tumor cells themselves expressed the highest levels of MCP-1 (Figure 1C,D). Likewise, in peripheral blood, CD11b+ Ly6C^high^ inflammatory monocytes expressed higher levels of CCR2 than anti-inflammatory monocytes or neutrophils (Figure S6). Analogous data were obtained using Lewis lung adenocarcinoma cells (Figure S7).

### 2.3. Lack of Tumor Growth in CCR2KO Mice

**Figure 2:**
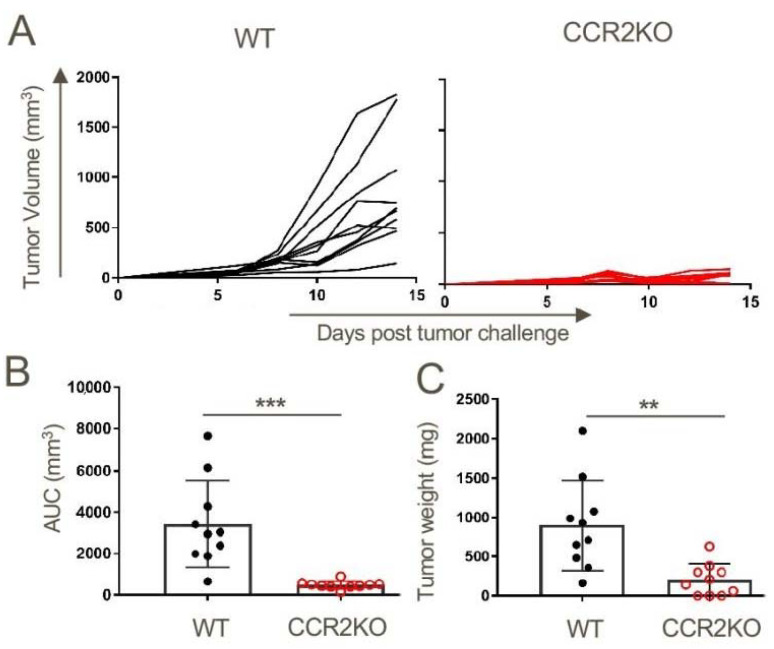
Growth of subcutaneously transplanted TC1 mouse lung tumors is dependent on CCR2 signaling. TC1 tumor cells (1 × 10^6^) were injected subcutaneously into WT and CCR2KO mice. (**A**) Tumors were measured biweekly until they were harvested on day 14 post-tumor inoculations, when (**B**) tumor volumes and (**C**) tumor weights were determined; ** *p* < 0.1 and *** *p* < 0.001, *n* = 10/group. Data are representative of three independent experiments.

Given the prominent MDSC expression of CCR2, we assessed TC1 tumor growth in C57BL/6 mice lacking CCR2 (CCR2KO). Compared to WT controls, tumor growth in CCR2RKO mice was significantly inhibited (Figure 2A), with reductions in tumor volumes (Figure 2B) and tumor masses (Figure 2C).

### 2.4. Host CCR2 Deletion Led to Markedly Reduced MDSCs but Increased Activated CD8+ T Cells at Tumor Sites

**Figure 3:**
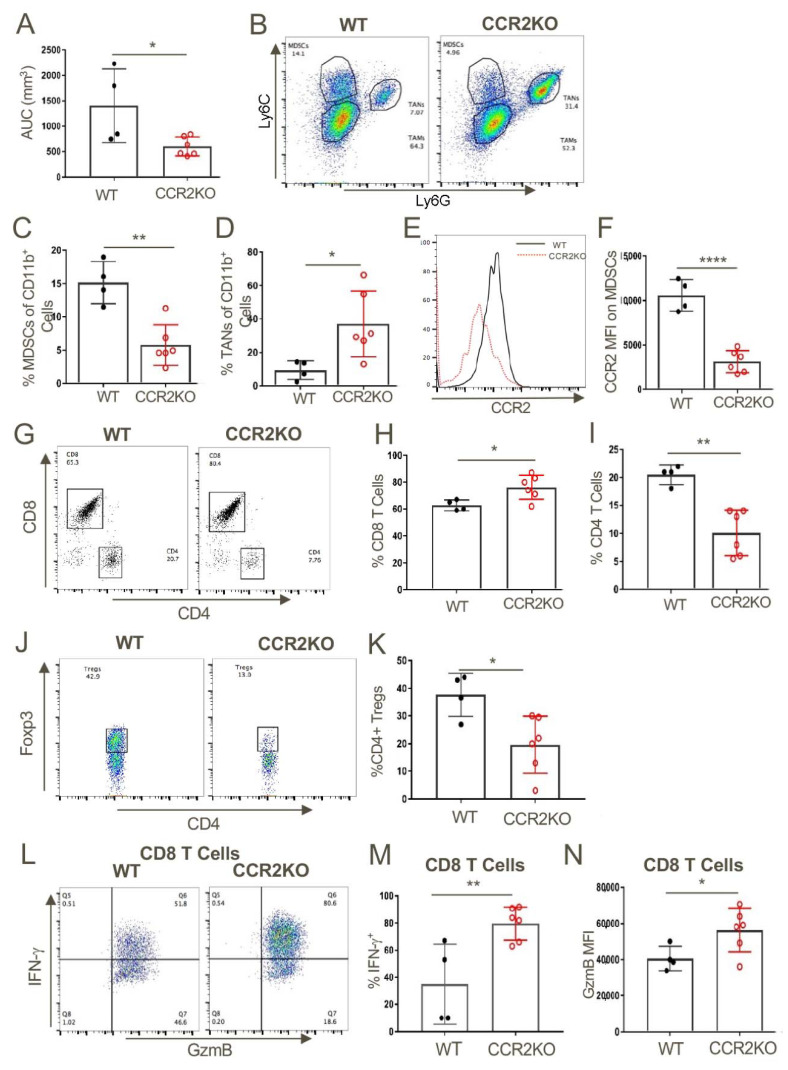
Genetic ablation of CCR2 reduced MDSC and Treg infiltration and increased intratumoral accumulation of activated CD8+ T cells. (**A**) TC1 tumor cells (1 × 10^6^) were injected subcutaneous (s.c.) in WT and CCR2KO mice and tumors were measured until day 14. Analysis of the intratumoral CD11b+ myeloid populations showed a significant decrease in MDSCs (**B**,**C**) and an increase in TAN (**B**,**D**). MDSCs from CCR2KO mice expressed negligible levels of CCR2 (**E**,**F**) as compared to MDSCs in tumors from WT mice. Tumors in CCR2KO mice also showed a higher frequency of CD8+ T cells (**G**,**H**) and fewer CD4+ T cell (**G**,**I**) and Tregs (**J**,**K**) as compared to WT control. Intratumoral CD8+ T cells from CCR2KO mice expressed significantly higher levels of IFN-γ (**L**,**M**) and GzmB (**N**) upon restimulation; * *p* < 0.05, ** *p* < 0.01, **** *p* < 0.001. Data are representative of three independent experiments.

We next assessed what effects CCR2 deletion had on anti-tumor immune responses by harvesting tumors at 14 days post-injection, preparing single-cell suspensions and flow cytometric analysis (Figure 3). The reduced tumor growth in CCR2KO mice (Figure 3A) was associated with significantly decreased numbers of MDSCs but significantly increased numbers of TAN (Figure 3B–D). As expected, expression of CCR2 on MDSCs in CCR2KO was markedly reduced compared to MDSCs in WT mice (Figure 3E,F). CCR2 deletion also led to increased CD8+ T cell infiltration but reduced accumulation of CD4 T cells (Figure 3G–I), including reduced numbers of CD4+Foxp3+ T-regulatory (Treg) cells (Figure 3J,K). Analysis of intracellular cytokine expression showed that compared to corresponding WT mice, the tumor-infiltrating CD8+ T cells of CCR2KO mice produced increased amounts of IFN-γ and granzyme-B (Figure 3L,M).

### 2.5. Importance of the CCR2/MCP-1 Pathway for Recruitment of Inflammatory Monocytes

**Figure 4:**
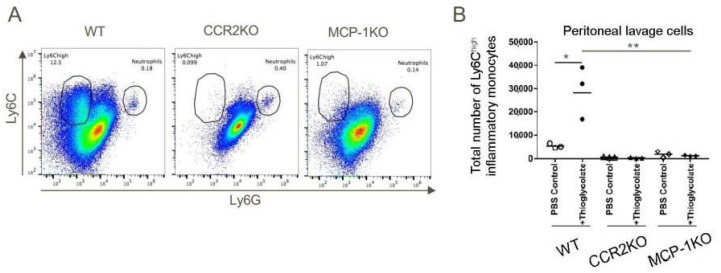
The MCP-1/CCR2 chemokine/chemokine receptor pathway axis has an indispensable role in the migration of CD11b+Ly6C^high^ cells to sites of inflammation. WT, CCR2KO and MCP1KO mice were injected with thioglycolate broth and 48 h later peritoneal lavage cells were analyzed by flow cytometry. (**A**,**B**) CD11b+Ly6C^high^Ly6G^low^ inflammatory monocytes failed to migrate in response to thioglycolate-elicited inflammation in CCR2KO and MCP-1KO mice; * *p* < 0.05 and ** *p* < 0.01 (*n* = 3/group). Data are representative of three independent experiments.

We next sought to create a system to test and validate the effects of a CCR2 antagonist on recruitment of inflammatory monocytes, given potential translational significance for tumor therapy. We first injected WT, CCR2KO and MCP1KO mice with 4% sterile thioglycolate broth and 48 h later, the total numbers of cells in peritoneal lavages were counted and the influx of inflammatory monocytes analyzed by flow cytometry. These studies showed potent recruitment of CD11b^+^Ly6C^high^ inflammatory monocytes to the peritoneum in WT mice in response to thioglycolate elicited inflammation, whereas peritoneal recruitment of these cells was largely abolished in CCR2KO or MCP-1KO mice (Figure 4).

**Figure 5:**
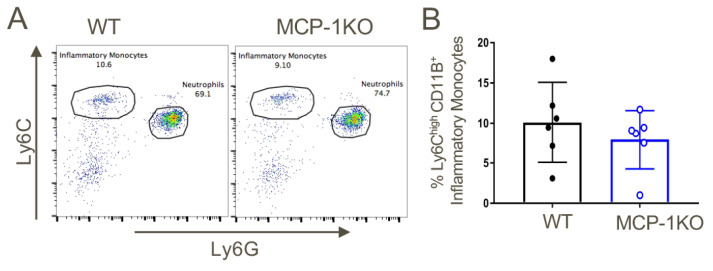
Analysis of baseline CD11b+Ly6C^high^ inflammatory monocytes in MCP-1KO mice. (**A**) Similar proportions of CD11b+Ly6C^high^ inflammatory monocytes at baseline within the peripheral blood of MCP-1KO vs. WT mice (*n* = 6/group). (**B**) Quantitative analysis (mean ± SD). Data are representative of three independent experiments.

Given our earlier observation of a paucity of splenic CD11b+Ly6C^high^ inflammatory monocytes in CCR2KO vs. WT mice housed under baseline conditions (Figure S3), their lack of recruitment to the peritoneum in response to thioglycolate could simply have reflected their relative deficiency. We therefore next checked the numbers of CD11b+Ly6C^high^ inflammatory monocyte at baseline in MCP-1KO mice. In contrast to their low numbers in CCR2KO mice, the proportions of CD11b+Ly6C^high^ inflammatory monocytes in MCP-1KO mice were comparable to that of WT mice (Figure 5).

**Figure 6:**
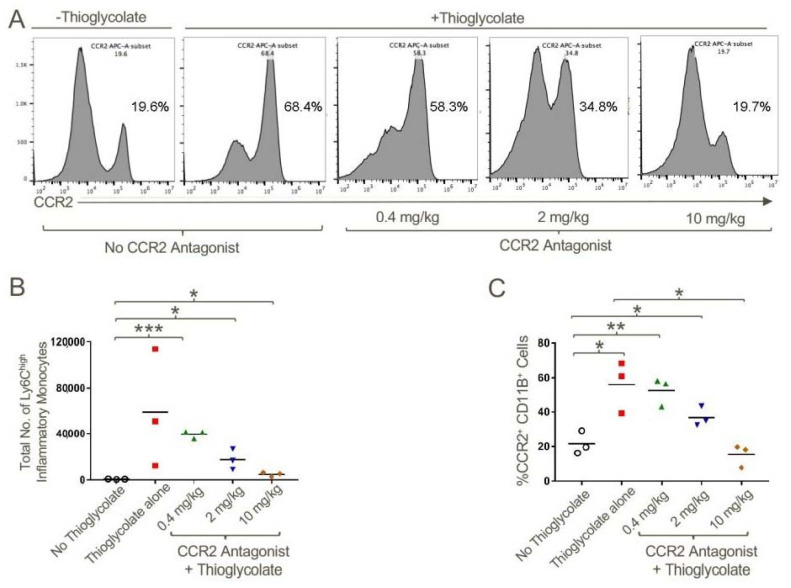
CCR2 antagonist inhibited the migration of CCR2+CD11b+Ly6C^high^ inflammatory monocytes to the site of inflammation. WT mice were injected with 4% sterile thioglycolate broth or vehicle control. Mice were treated with vehicle alone or CCR2 antagonist twice daily at a concentration of 0.4, 2 or 10 mg/kg for 48 h post-thioglycolate injections. Total number of cells in peritoneal lavage were counted. (**A**) Representative histogram plot demonstrating the increased percentage of CCR2+ cells in the peritoneal cavity in response to thioglycolate induced inflammation and abrogation of this effect upon CCR2 antagonist treatment in a dose-dependent manner. (**B**) Quantification of total number of Ly6C^high^ inflammatory monocytes and (**C**) CD11b+CCR2+ cells in the peritoneal lavage in response to the treatments noted; * *p* < 0.05, ** *p* < 0.01 and *** *p* < 0.001; *n* = 3/group and data are representative of three independent experiments.

Lastly, to assess whether a potent CCR2 small-molecule antagonist (IC50 ~5 nM) [28] could inhibit CCR2-dependent recruitment of inflammatory cells, we injected thioglycolate into the peritoneal cavities of WT mice and treated mice with CCR2 antagonist or carrier alone. We found that the compound decreased recruitment of CCR2+CD11b+Ly6C^high^ inflammatory monocytes in a dose-dependent manner (Figure 6).

Collectively, these data showed that we could use a commercially available CCR2 antagonist to assess the effects of CCR2 inhibition on MDSC recruitment and syngeneic tumor cell growth in WT mice. However, before undertaking those studies, given reports of CCR2 expression by T cells, including Foxp3+ Tregs [29,30], we felt it prudent to consider whether CCR2 targeting might also affect the activation and proliferation of CD4+ and CD8+ effector T cells, as well as the suppressive functions of CD4+Foxp3+ Treg cells.

### 2.6. CCR2KO Mice Had Normal CD4+ and CD8+ T cells, Including Fully Functional CD4+Foxp3+ Treg Cells

**Figure 7:**
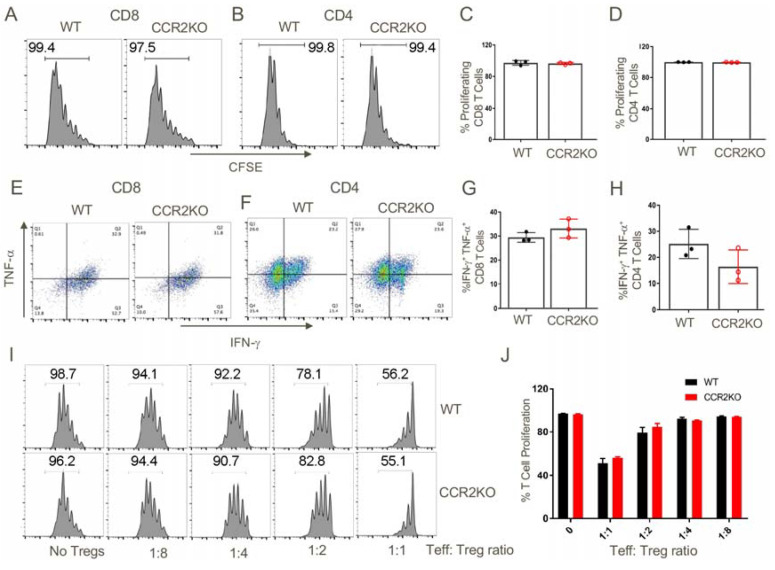
Genetic ablation of CCR2 did not affected the ability of T cells to proliferate and produce inflammatory cytokines or the suppressive capability of Foxp3+ Tregs. CFSE-labelled T cells (70 × 10^6^) from the spleens and lymph nodes of WT or CCR2KO C57BL/6 mice were injected i.v. into B6D2/F1 mice. After 3 days, spleens were harvested from the adoptively transferred B6D2/F1 mice and analyzed for the extent of proliferation of transferred T cells and their ability to produce inflammatory cytokines. (**A**–**D**) In vivo proliferation WT and CCR2KO CD8+ T cells and CD4+ T cells were comparable. (**E**–**H**) CD8 and CD4 T cells from WT and CCR2KO mice were equally capable of secreting inflammatory cytokines (TNF-α, IFN-γ) upon restimulation in vitro. (**I**,**J**) WT and CCR2KO CD4+ Foxp3+ Tregs suppressed conventional CD4 T cell proliferation to a similar extent. Data are representative of three independent experiments.

We assessed the effects of CCR2 gene deletion on T cell alloactivation, proliferation and cytokine production in a parent → F1 model by adoptively transfering CCR2RKO or WT T cells from C57BL/6 mice into F1 (C57BL/6 × DBA/2) recipients. We found that CCR2RKO mice had comparable T cell proliferative and functional responses to WT mice, as well as showing comparable Treg suppressive function (Figure 7).

### 2.7. CCR2 Antagonist Inhibited the Growth of Syngeneic Tumor Cells In Vivo

**Figure 8:**
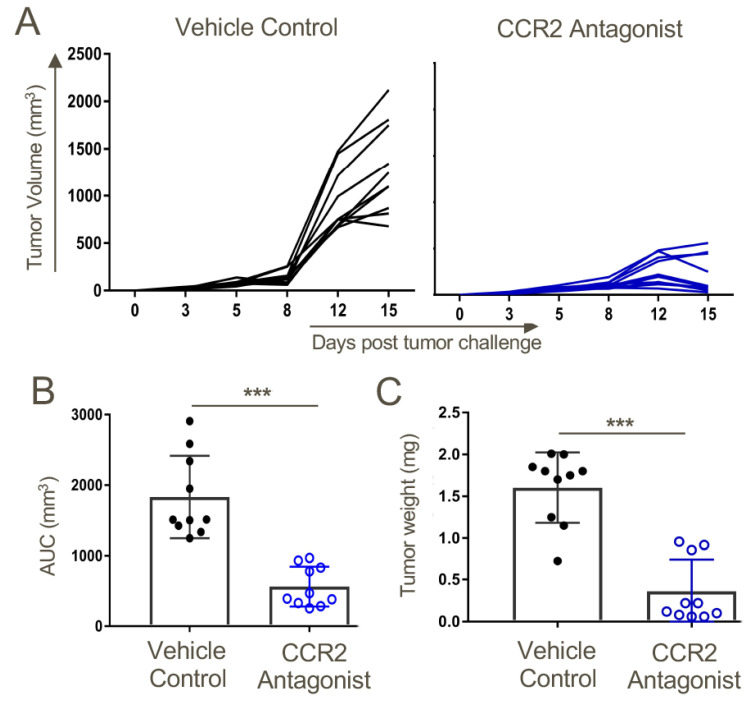
Therapy with a CCR2 antagonist inhibited tumor growth in syngeneic WT mice. Mice were transplanted s.c. with 1.2 × 10^6^ TC1 tumor cells. On day 3 post-tumor inoculations, mice were randomly distributed to obtain similar (~20 mm^3^) average tumor sizes across both groups before starting CCR2 antagonist treatment. Mice were treated twice daily with a 10 mg/kg dose of CCR2 antagonist or vehicle control until the day of harvest on day 15. (**A**) Tumor measurements in response to CCR2 antagonist or vehicle control treatments. (**B**) Area under the curve analysis. (**C**) Tumor volumes on the day of tumor harvest; *n* = 10/group and *** *p* < 0.001. Data are representative of three independent experiments.

Seeking data of potential translational significance, we returned to our original immuno-oncology focus and assessed the effects of the CCR2 antagonist on the growth of TC1 tumor cells in syngeneic WT mice. We found the CCR2 antagonist markedly impaired tumor growth (Figure 8).

### 2.8. Effects of the CCR2 Antagonist on Tumor-Infiltrating Myeloid Cells

**Figure 9:**
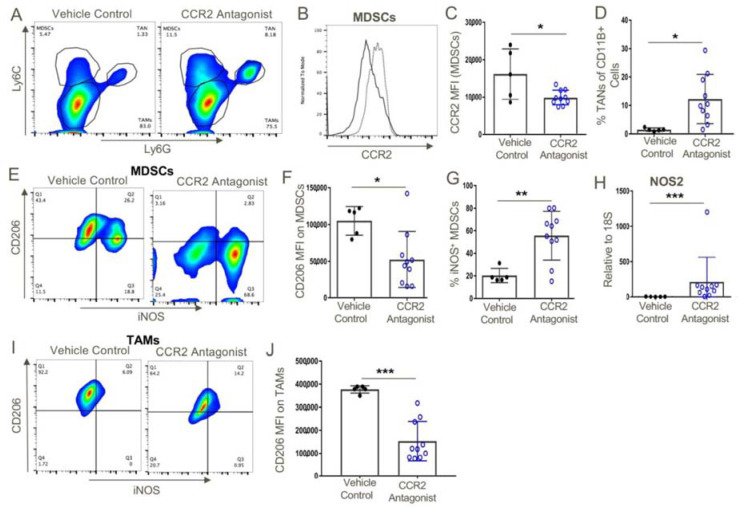
MDSCs within CCR2 antagonist-treated mice expressed significantly lower levels of CCR2 and both MDSCs and TAMs expressed an M1 phenotype as opposed to the M2 phenotype of vehicle control-treated mice. (**A**) Intratumoral analysis post-therapy with vehicle control or CCR2 antagonist (10 mg/kg/d) showed the presence of MDSCs within the tumor microenvironment. (**B**,**C**) However, these MDSCs expressed significantly lower levels of CCR2 as opposed to vehicle control-treated mice. (**D**) CCR2 antagonist treatment also resulted in a significant increase in the frequency of TANs. (**E**–**H**) MDSCs and (**I**,**J**) TAMs from CCR2 antagonist-treated mice displayed an M1 phenotype as indicated by their downregulation of CD206, and the MDSCs displayed higher iNOS transcript and protein levels as compared to MDSCs from vehicle control-treated mice; * *p* < 0.05, ** *p* < 0.01, and *** *p* < 0.001 (*n* = 10/group). Data are representative of three independent experiments.

Given our data on how CCR2 gene deletion reduced MDSC and Treg infiltration and increased intratumoral accumulation of activated CD8+ T cells (Figure 3), we undertook a corresponding analysis of cells present within the tumor microenvironment of WT mice receiving vehicle or CCR2 antagonist therapy. Given its efficacy in blocking CCR2+ cell recruitment in the thioglycolate studies (Figure 6), we expected the compound would decrease MDSC numbers within tumor-bearing WT mice. This was not the case but rather the MDSCs that were still present expressed lower levels of CCR2 and had lower levels of CD206 and higher levels of inducible nitric oxide synthase (iNOS) than MDSCs in control-treated mice (Figure 9). Additionally, TAMs in CCR2 antagonist mice had decreased expression of CD206, a marker of an M2 phenotype. Hence, use of the CCR2 antagonist induced cells with more of an M1 phenotype compared to the M2 phenotype associated with MDSCs and TAMs of tumor-bearing WT mice.

### 2.9. Effects of the CCR2 Antagonist on Tumor-Infiltrating T Cells

**Figure 10:**
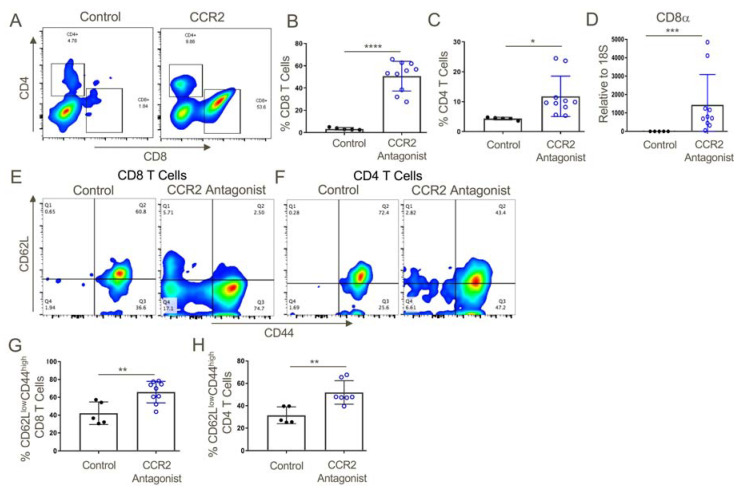
CCR2 antagonist treatment resulted in greater intratumoral infiltration of activated T cells as compared to vehicle control-treated mice. (**A**,**B**) Tumors from CCR2 antagonist-treated mice exhibited significantly increased infiltration of CD8+ and (**A**,**C**) CD4+ T cells, and also contained increased transcript levels of (**D**) CD8α as compared to vehicle control-treated tumors. Additionally, both (**E**,**G**) CD8+ and CD4+ (**E**,**H**) T cells from CCR2 antagonist-treated mice displayed a more activated phenotype (increased CD44 and decreased CD62L) compared to T cells from the control group; *n* = 5–9/group, * *p* < 0.05, ** *p* < 0.01, *** *p* < 0.005, and **** *p* < 0.001. Data are representative of three independent experiments.

**Figure 11:**
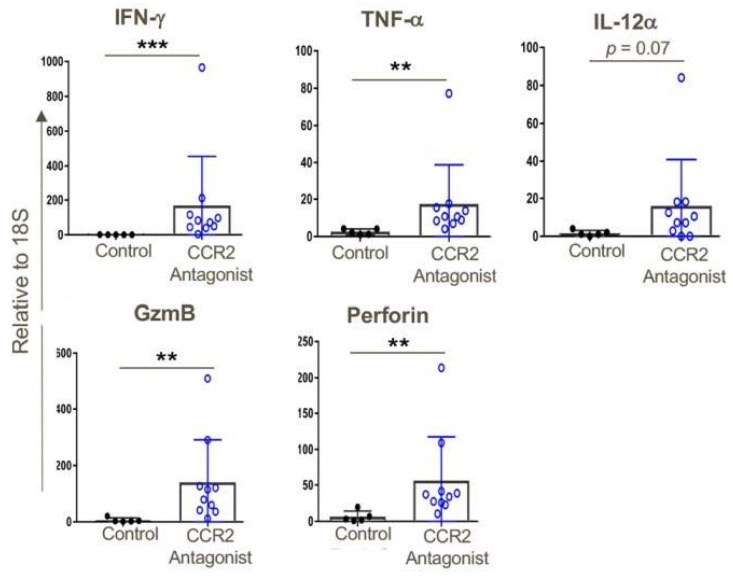
CCR2 antagonist treatment resulted in greater intratumoral expression of pro-inflammatory genes compared to that of tumors in vehicle-treated mice; qPCR data, ** *p* < 0.01, *** *p* < 0.001, and *n* = 5–10 mice/group. Data are representative of three independent experiments.

Consistent with the effects of CCR2 gene deletion on anti-tumor responses (Figure 3), use of a CCR2 antagonist significantly increased CD8 T cell infiltration and to a lesser extent also boosted CD4+ T cell infiltration, too, and both intratumoral T cell populations had the phenotype of effector/memory T cells (CD44^hi^ CD62L^low^) (Figure 10). In conjunction with the influx of activated T cells, increased expression of several pro-inflammatory cytokines was observed, including IFN-γand TNF-α, and increased expression of the cytolytic granules, granzyme-B and perforin, commonly associated with the actions of effector CD8+ T cells (Figure 11). We also assessed effects of CCR2 antagonist therapy of memory responses by re-challenging mice with a second injection of tumors 30 days after the previous experiment had ceased. We found that mice that had rejected the TC1 tumors in response to therapy with a CCR2 antagonist in the first round of tumor studies had developed potent memory responses and showed complete inhibition of growth of tumors upon re-challenge (Figure S8).

### 2.10. Increased Cytotoxicity of T Cells from CCR2 Antagonist-Treated Mice

**Figure 12:**
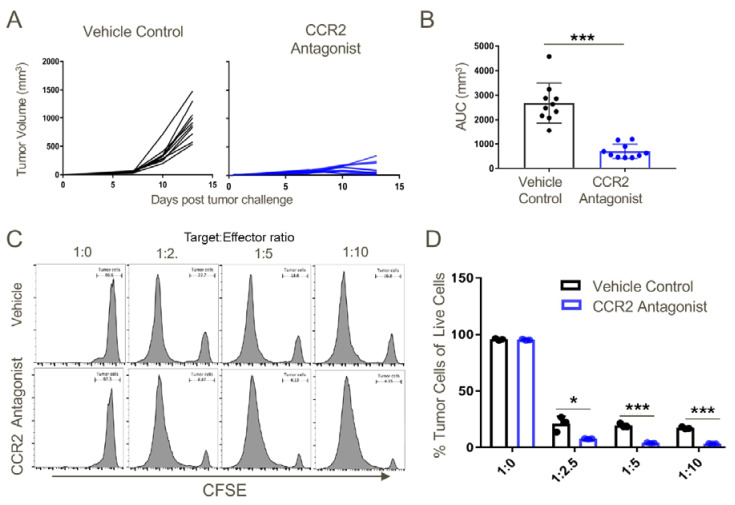
T cells derived from tumor-draining lymph nodes of CCR2 antagonist-treated mice displayed greater cytotoxic potential against TC1 tumor cells as compared to the tumor-draining lymph node cells of vehicle-treated mice. Transplanted TC1 tumors were treated with CCR2 antagonist or vehicle control when they reached ~50 mm^3^ until the day of harvest (Day 14). On the day of harvest, lymph node cells were cocultured with 1 × 10^5^ CFSE-labelled TC1 tumor cells at indicated effector: target ratios. (**A**) Tumor growth curves in response to vehicle control or CCR2 antagonist treatment. (**B**) Area under the curve analysis, (**C**) representative histograms and (**D**) cumulative data showing the greater cytotoxic potential of CCR2 antagonist-treated tumor-draining lymph node T cells against TC1 tumor cells at indicated target:effector ratios as compared to T cells from tumor lymph nodes in mice receiving vehicle control; * *p* < 0.05, *** *p* < 0.001, and *n* = 3/group. Data are representative of three independent experiments.

In further studies, the effects of therapy with the CCR2 antagonist on anti-tumor cytotoxicity was determined by isolating T cells from draining lymph nodes at day 14 post-TC1 injection. We found that the CCR2 antagonist markedly impaired tumor growth (Figure 12A,B) and significantly increased the T cell cytotoxicity (Figure 12C,D).

### 2.11. Production of MCP-1 by Tumor Cells Rather Than Host Cells Promotes Tumor Growth

**Figure 13:**
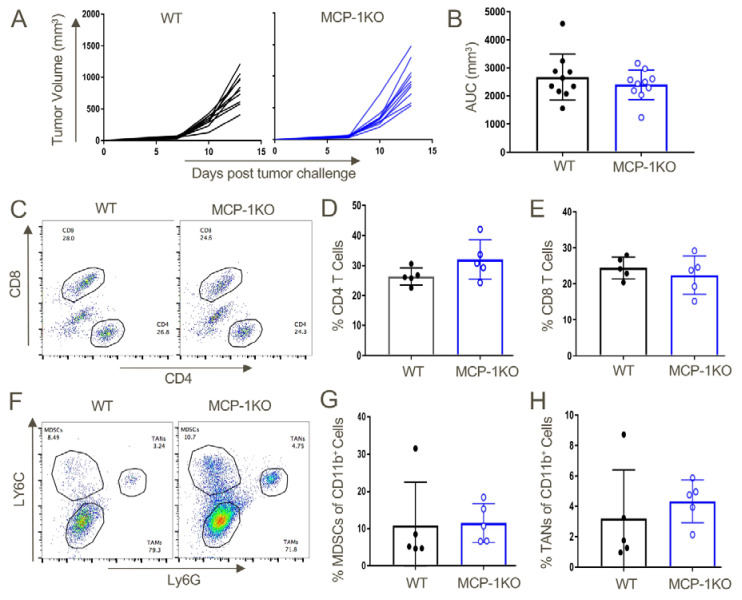
Tumor-derived MCP-1 appears indispensable for the growth of TC1 tumors and recruitment of MDSCs to the tumor microenvironment. TC1 tumors were transplanted in WT or MCP-1KO mice and monitored until day 13 post-injection, when they were harvested and analyzed by flow cytometry. (**A**) Tumor growth curves in WT and MCP-1KO mice and (**B**) area under the curve analysis. Intratumoral T cell analysis from WT and MCP-1KO mice revealed comparable (**C**,**D**) CD4 and CD8 (**C**,**E**) T cell populations. Myeloid lineage analysis also displayed similar overall frequencies of (**F**,**G**) MDSCs and (**F**,**H**) TANs in tumors grown in WT vs. MCP-1KO mice (*n* = 5/group and data are representative of three independent experiments).

Lastly, given the importance of CCR2+ MDSC recruitment to promoting tumor growth in our studies, we assessed whether production of MCP-1/CCL2 was host derived or tumor derived by testing syngeneic tumor growth in WT vs. MCP-1KO mice. We found comparable growth in WT vs. MCP-1KO mice (Figure 13A,B), as well as comparable recruitment of CD4+ and CD8+ T cells (Figure 13C–E), and MDSCs and TAN (Figure 13F–H). Hence, we conclude that CCR2-depenednt effects observed in our studies likely reflect the production of MCP-1 by tumor cells rather than host cells.

## 3. Discussion

Monocytes express four chemokine receptors relevant to their migration to inflammatory sites, CCR1, CCR2, CCR3 and CCR5 [31]. From an evolutionary perspective, CCR2 is the oldest of these receptors and it is thought that it gave rise to the other three as a result of gene duplication [32]. Such duplication has led to decades of analysis in efforts to unravel the complexity of chemokine/chemokine receptor expression and signaling and has often resulted in frustrations over what was interpreted as chemokine/chemokine receptor redundancy [33,34]. However, recent studies have highlighted CCR2 as the primary driver of myelomonocytic recruitment during inflammation [35]. In addition, the importance of chemokines and their receptors in regulating anti-tumor immunity and tumor metastases were recently reviewed [36,37,38], including the dominant role of CCR2 in promoting recruitment of monocytes with pro-tumorigenic and pro-metastatic activities. Consistent with this, ongoing clinical trials have reported encouraging results of CCR2 inhibitors, especially as combination therapies, in patients with pancreatic cancers, hepatocellular carcinomas and ovarian cancers [37].

The current study arose from a desire to further understand the complex interactions occurring within the tumor microenvironment and how these events might be targeted therapeutically. We focused on analyzing the role of CCR2-MCP-1 signaling in the recruitment of CCR2+ MDSCs into the tumor microenvironment using syngeneic, immunocompetent mice. Tumor cells recruited CCR2+ MDSCs through their expression of high levels of MCP-1/CCL2, the ligand for chemokine CCR2. Genetic and pharmacological intervention of CCR2-MCP-1 signaling significantly decreased tumor growth in conjunction with increased effector cytotoxic CD8+ T cells. These effects were MDSC dependent as both WT and CCR2KO CD4+ and CD8+ T cells displayed comparable ability to proliferate and produce effector cytokines. Additionally, the ability of Tregs to suppress effector T cell proliferation was comparable between WT and CCR2KO mice.

Surprisingly, the frequency of tumor-associated MDSCs was not decreased in CCR2 antagonist-treated mice, but the MDSCs now displayed significantly lower levels of CCR2 expression and had an M1 phenotype. Taken together, ablation of CCR2-MCP1 signaling boosts anti-tumor efficacy by preventing the tumor infiltration of immunosuppressive CCR2+ M2 MDSCs and by promoting cytotoxic T cell responses. CCR2 targeting may be of considerable translational significance given that a second CCR2+ cell population, CCR2+ TAMs, can mediate immune evasion by inducing PD-L2 expression by tumors [39]. Hence, there may be clinical utility by combining currently approved anti-PD-1 mAb with targeting of CCR2/MCP-1 so as to overcome multiple mechanisms of tumor immune evasion [27].

The basis for the CCR2 antagonist leading to an M1 phenotype of MDSCs present at the tumor site in our studies is currently unknown, though there are some clues from the literature. Constitutive MCP-1 production by monocytes, dendritic cells and endothelial cells is counteracted by ongoing uptake and internalization by CCR2+ cells [40]. Hence, in CCR2RKO mice or during treatment with a CCR2 antagonist, including the compound used in the current study, blood levels of MCP-1 are markedly elevated [40]. MCP-1 is typically linked with M2 responses but its role in macrophage polarization may vary by context, given its association with M1 macrophages in inflammatory bowel disease, rheumatoid arthritis, multiple sclerosis and type 2 diabetes [41]. Further studies are required to ascertain whether raised levels of MCP-1 contributed to the M1 phenotype of CCR2+ MDSCs present within tumors of mice treated with the CCR2 antagonist, or whether simply CCR2 blockade induced this phenotype, as has been shown can occur in vitro using murine macrophage cell lines cultured in the presence of CCR2 antagonists [42]. Likewise, additional studies are required to determine whether insufficient inhibition of CCR2-mediated migration likely explains the persistence of CCR2+ cells within tumors or whether additional chemokine/chemokine receptor pair(s) were responsible for recruitment of these cells to tumors despite use of the CCR2 antagonist.

In addition to a change to an M1 phenotype of the CCR2+ MDSCs in mice treated with the CCR2 antagonist, we were surprised to find considerable CCR2 expression by the tumor cells themselves (Figure 1). Relatively little is known concerning the functions of CCR2 on tumor cells, though one group reported that the binding of MCP-1 to CCR2 can induce the survival and migration of breast cancer cells [43]. In addition, just prior to submission of our paper, CCR2 expression by murine breast cancer cells was shown to support immune evasion by inhibiting dendritic cell infiltration and maturation, suppressing cytotoxic T cell activity, decreasing MHC class I expression and upregulating PD-L1 expression [44]. If these data can be confirmed beyond breast cancer models in mice, the utility of targeting CCR2 may well extend beyond its effects on immunosuppressive cells in the tumor microenvironment to encompass direct effects on tumor cells themselves.

## 4. Materials and Methods

### 4.1. Animals

We purchased C57BL/6 (H-2^b^), CCR2KO mice (B6.129S4-CCR2^tm1Ifc/J^, Stock No. 004999) and MCP-1KO mice (B6.129S4-CCL2^tm1Rol/J^, Stock No. 004434) on a C57BL/6 background, and B6D2F1/J (H-2^b/d^, Stock No. 100006) from The Jackson Laboratory (Bar Harbor, ME, USA). Mice were housed under pathogen-free conditions and were used at 5–8 weeks of age, unless specified. All studies were conducted in accordance with the GSK Policy on the Care, Welfare and Treatment of Laboratory Animals and used protocols approved by the Institutional Animal Care and Use Committee of the Children’s Hospital of Philadelphia (IAC 2017-001047, 12 December 2019, Waltham, MA, USA).

### 4.2. Antibodies

We purchased directly conjugated monoclonal antibodies for flow cytometry from Invitrogen (Waltham, MA, USA): CD4-PE, Clone GK1.5; CD11b-ef450, Clone M1/70; CD44-FITC, Clone IM7; CD45AF700, Clone 30-F11; CD206-APC, Clone MR6F3; FoxP3-ef450, Clone FJK-16S, IFN-γ-PE, Clone XMG1.2; GzmB-APC, Clone GB11; iNOS-PE, Clone CXNFT; live dead fixable dead cell stain kit, Catalogue No. L34959. BD Pharmingen (San Jose, CA, USA): CD3-AF700, Clone 17A2; CD4-Pacblue and perCPcy5.5, Clone RM4-5; CD8-FITC, perCPcy5.5, and PECY7, Clone 53-6.7; CD62L-PECY7, Clone MEL-14; H2Kd-PE, Clone 17A2; Ly6G-PE and percpcy5.5, Clone 1A8; Ly6C-APCCY7, Clone- AL-21; TNF-α PECY7, Clone MP6-XT22. Biolegend (San Diego, CA, USA): CD3-PECY7 Clone 17A2; B220-percpcy5.5, Clone RA3-6B2. R&D Systems: CCR2-APC, Clone 475301; CCL2-APC, Clone 123616. eBioscience (San Diego, CA, USA): H2Kd/Dd-ef450 Clone 34-1-25; CD4-APC, Clone GK1.5.

### 4.3. Cell Lines and Tumor Models

For mouse tumor studies, the TC1 tumor cell line was derived from C57BL/6 mouse lung epithelial cells, immortalized with HPV-16E6 and E7 and transformed with the c-Ha-ras oncogene [45]. TC1 tumor cells (1 × 10^6^) were transplanted subcutaneously in a total volume of 100 µL in WT, CCR2 KO or MCP-1 KO mice using the right flank of each mouse except for the re-challenged experiment when mice were re-challenged with 1 × 10^6^ TC1 tumor cells on the left flank 30 days post-primary TC1 tumor rejection. Additional studies were performed using Lewis lung carcinoma cells (ATCC). Tumor volumes were measured by using a standard formula (long diameter × short diameter × short diameter/2) and recorded in mm^3^ until the indicated day of tumor harvest or experimental endpoint.

### 4.4. Single-Cell Preparations

TC1 tumors were harvested from mice post-euthanization and placed in 1XHBSS media (Gibco, Gaithersburg, MD, USA, Catalogue No. 24020-117). Tumors were chopped into small pieces (2–4 mm) using scissors and forceps and transferred into gentleMACS tube (Miltenyi Biotech, San Diego, CA, USA, Catalogue No. 130-096-334) in a total volume of 5 mL comprised of 2.5 mL 1XHBSS + 2.5 mL tumor dissociation kit (Miltenyi Biotech, Catalogue No. 130-096-730). Dissociated tumor tissues were incubated at 37 °C at 200 rpm for 40 min. Post-incubation, a single-cell suspension was prepared by passage through 70 µM strainers (ThermoFisher, San Diego, CA, USA, Catalogue No. 22363548). Cells were pelleted and RBCs were lysed by adding 3 mL of 1X RBS lysis buffer (eBioscience, Catalogue No. 00-4333-57). Cells were finally resuspended in 1 mL PBS and further processed for flow cytometric analysis. To obtain single-cell suspensions from spleen and lymph nodes, tissues were harvested, directly crushed through the strainers, RBCs were lyzed and cells processed for flow cytometry. Peripheral blood cell populations were analyzed by retro-orbital bleeding of mice, lysis of RBCs and processing the remaining cells for flow cytometry.

### 4.5. Thioglycolate-Induced Peritonitis Model

A volume of 1 mL sterile of freshly prepared 4% thioglycolate broth (Sigma Aldrich, St. Loius, MO, USA, Catalogue No. 70157) in double distilled water was injected intraperitoneally in WT, CCR2KO or MCP-1KO mice. At 48 h post-thioglycolate injections, 10 mL of ice-cold sterile PBS was injected into the peritoneal cavity of mice utilizing a 26-G needle. Peritoneal lavage was retrieved using an 18-G needle connected to a 10 mL syringe. The total volume of retrieved lavage was recorded and processed for flow analysis. To evaluate the effective dose response studies, a commercially available small-molecule CCR2 antagonist [28], was injected (10 mg/kg) 30 min before thioglycolate broth injections at the indicated doses.

### 4.6. Suppression, Proliferation, and Cytokine Release Assays

Tregs and conventional non-Treg CD4 T cells were isolated from WT or CCR2 KO mice using a Treg isolation kit (Miltenyi Biotech, Catalogue No. 130-091-041). The 5 × 10^4^ CFSE labelled non-Treg conventional CD4 T cells were stimulated with CD3 mAb (1 µg/mL) in the presence of 5 × 10^4^ irradiated T cell-depleted splenocytes and varying ratios of Tregs, as indicated, for 72 h. The suppression ability of WT and CCR2 KO Tregs cell was compared by analyzing CFSE dilution.

In vivo proliferation of WT and CCR2 KO T cells was determined in a GVHD model that was induced by adoptively transferring 70 million CFSE-labelled total cells derived from spleen and lymph nodes of WT or CCR2 KO C57BL/6 mice into B6D2/F1 mice that were heterozygous for B6 and D2 alleles at all genomic loci. In vivo T cell proliferative of WT and CCR2 KO T cells were compared by CFSE dilution using flow cytometry.

Pro-inflammatory cytokine (IFN-γ and TNF-α) secretion capability of WT and CCR2KO T cells was compared by re-stimulating the splenocytes (1 × 10^6^) of B6D2/F1 mice post-adoptive transfer of WT or CCR2KO cells using a stimulation cocktail (eBioscience, Cell Stimulation Cocktail 500×, Catalogue No. 00-4975-93, plus protein transport inhibitor Monensin, ×1000, Cat# 420701, Biolegend) for 5 h at 37 °C. This was followed by surface staining, fixing and permeabilizing the cells using a fixation/permeabilization solution kit (BD Pharmingen, Catalogue No. 554714) and eventually performing intracellular staining. Additionally, proinflammatory cytokine production by CD8 T cells derived from tumors extracted from WT and CCR2KO mice was also compared using above mentioned method.

### 4.7. Cell Cytotoxicity Analysis Against TC1 Tumor Cells

Cells derived from tumor-draining lymph nodes of mice treated with CCR2 antagonist (10 mg/kg, twice daily) or vehicle control were co-cultured with in vitro-cultured and CFSE-labelled (2.5 µM) 1 × 10^5^ TC1 tumor cells in U-bottom 96-well plate at the indicated ratios for 24 h at 37 °C. Percentage tumor cell killing was determined using flow cytometry.

### 4.8. Real-Time qPCR

Tumors were dissected from tumor bearing mice that were treated with either CCR2 antagonist or vehicle control and were flash frozen in liquid nitrogen. RNA was isolated using a RNeasy kit (Qiagen, San Diego, CA, USA) and its integrity and quantity was analyzed by NanoDrop (ND-1000) and Nanochip 2100 Bioanalyzer (Agilent Technologies, San Diego, CA, USA). RNA was reverse transcribed to cDNA (Applied Biosystems, San Diego, CA, USA) and Taqman primer and probe sets were utilized for qPCR execution. Data were normalized to endogenous 18 s rRNA and relative fold expression was determined.

### 4.9. Statistical Analyses

Data in this study were analyzed using GraphPad Prism 7 software and presented as the mean ± SD. The tumor growth rate over a period of time was calculated using area under the curve (AUC) analysis and differences between multiple groups were compared by one-way ANOVA with Turkey’s post-hoc test; *p* < 0.05 was considered a significant difference.

## 5. Conclusions

This study analyzed the role of CCR2-MCP-1 signaling in the recruitment of CCR2+ MDSCs into the tumor microenvironment in syngeneic, immunocompetent mice. Tumor cells recruited CCR2+ MDSCs through their expression of high levels of MCP-1/CCL2, the ligand for chemokine CCR2. Genetic and pharmacological intervention of CCR2-MCP-1 signaling significantly decreased tumor growth in conjunction with increased effector cytotoxic CD8+ T cells. These effects were MDSC dependent as both WT and CCR2KO CD4+ and CD8+ T cells displayed comparable ability to proliferate and produce effector cytokines. Additionally, the ability of Tregs to suppress effector T cell proliferation was comparable between WT and CCR2KO mice. Interestingly, though the frequency of MDSCs was not compromised in a CCR2 antagonist-treated group, MDSCs displayed significantly lower levels of CCR2 expression and attained an M1 phenotype. Taken together, ablation of CCR2-MCP1 signaling boosts anti-tumor efficacy by preventing the tumor infiltration of immunosuppressive CCR2+ M2 MDSCs and by promoting cytotoxic T cell responses, and may serve as a new therapeutic component in immuno-oncology.

## Supplementary Materials

The following are available online at https://www.mdpi.com/2072-6694/12/12/3723/s1, Figure S1: CCR2 gene deletion did not affect thymic T cell development, Figure S2: CCR2 gene deletion did not affect the frequencies of CD4+ or CD8+ T cells, including CD4+Foxp3+ Treg cells, in the periphery, Figure S3: CCR2 gene deletion significantly reduced the frequency of splenic Ly6C^high^ inflammatory monocytes, Figure S4: The frequency of CD11b+Ly6C^high^ inflammatory monocytes was comparable in the bone marrows of WT and CCR2KO mice, suggesting that the compromised frequency of these cells in the periphery was not attributable to a developmental defect in their production in CCR2KO mice, Figure S5: Gating strategy used to evaluate T cells and myeloid lineage populations in TC1 tumors, Figure S6: Blood CD11b+ Ly6C^high^ inflammatory monocytes expressed higher levels of CCR2 than anti-inflammatory monocytes or neutrophils, Figure S7: MDSCs associated with subcutaneously transplanted Lewis lung carcinoma cells expressed the highest levels of CCR2 whereas tumor cells were the main source of CCL2 (MCP-1), Figure S8: Mice that rejected the TC1 tumors in response to therapy with a CCR2 antagonist also developed memory responses.
