# Supplementary material for: The CCR2/MCP-1 Chemokine Pathway and Lung Adenocarcinoma
Source: Cancers (Basel). 2020 Dec 11;12(12):3723. doi: 10.3390/cancers12123723 (PMC7763565; doi:10.3390/cancers12123723)
Supplement: Supplementary file 1 [file cancers-12-03723-s001.pdf]

# Supplementary Material: The CCR2/MCP-1 Chemokine Pathway and Lung Adenocarcinoma

**Payal Mittal, Liqing Wang, Tatiana Akimova, Craig A. Leach, Jose C. Clemente, Mathew R. Sender, Yao Chen, Brandon J. Turenen and Wayne W. Hancock**

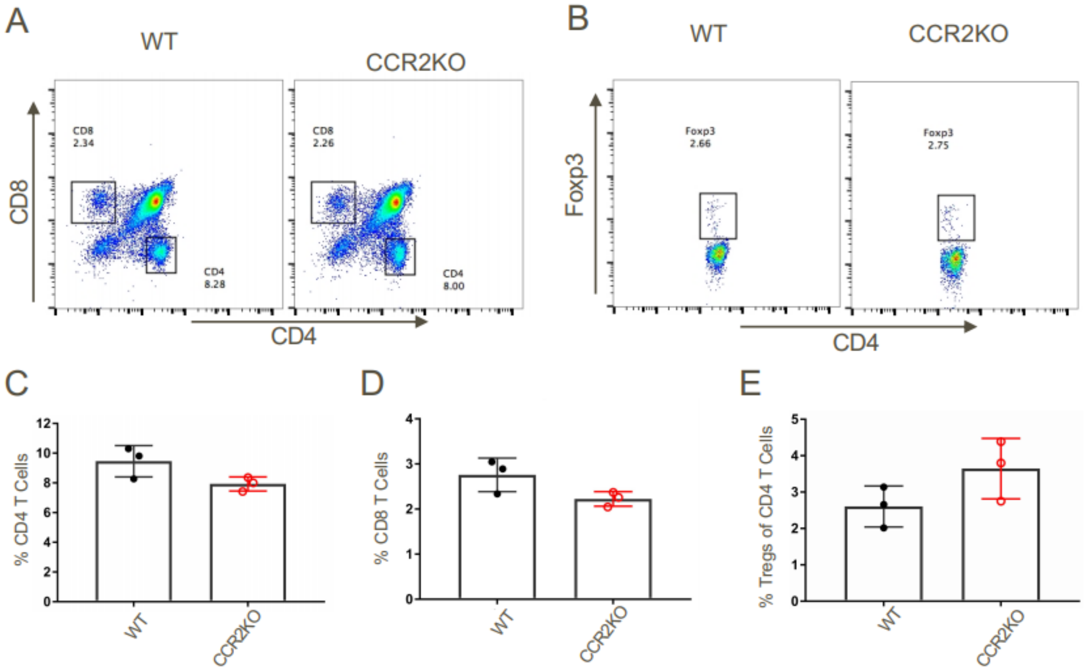


**Figure S1.** CCR2 gene deletion did not affect thymic T cell development. Analysis of T cell populations in the thymii of WT and CCR2KO mice displayed similar frequencies of (**A**,**C**) CD4, (**A**,**D**) CD8 and (**B**,**E**) CD4+Foxp3+ Treg cells ($n$ = 3/group).

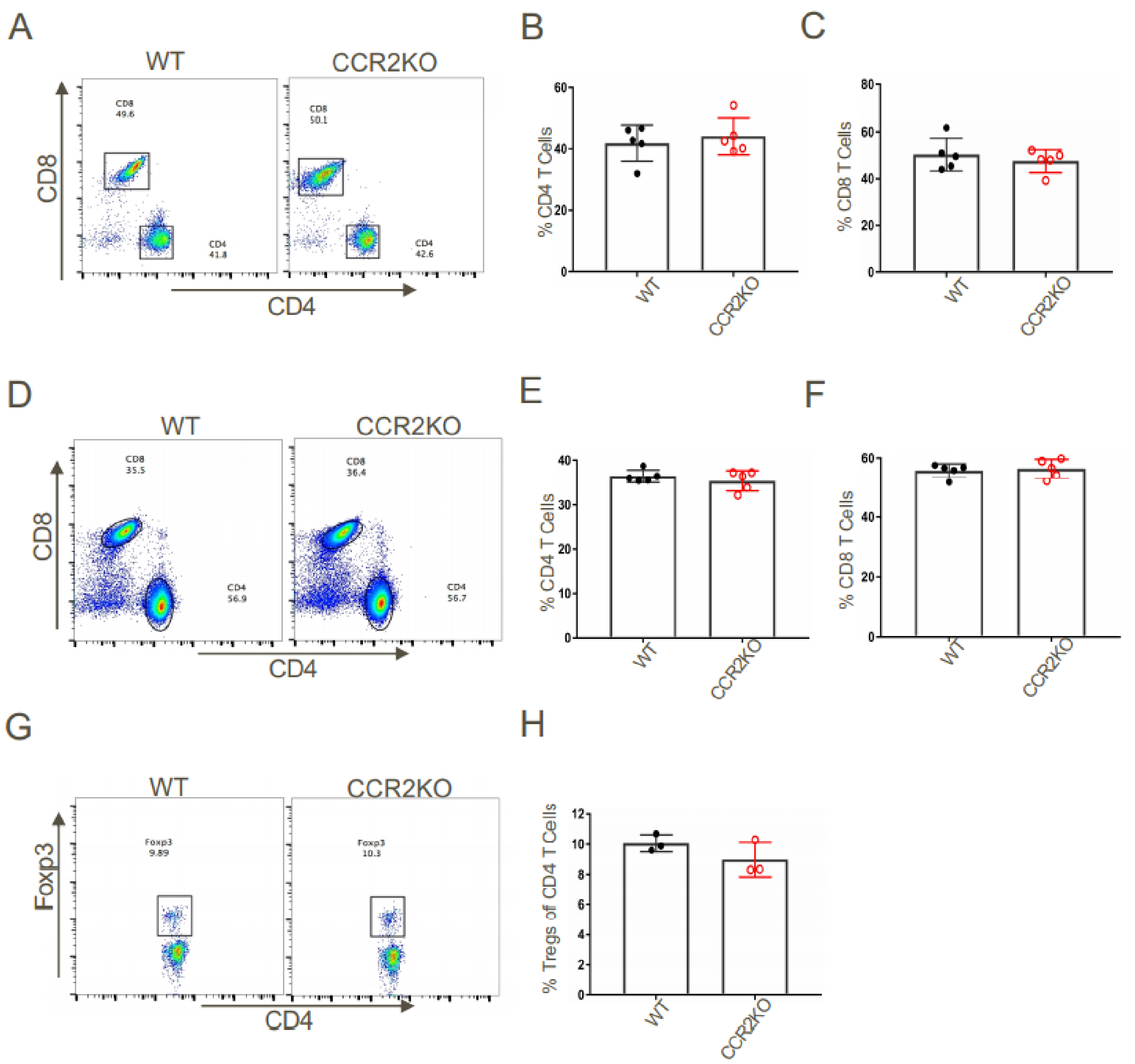


**Figure S2.** CCR2 gene deletion did not affect the frequencies of CD4+ or CD8+ T cells, including CD4+Foxp3+ Treg cells, in the periphery. Flow cytometric analysis showed comparable numbers of CD4 and CD8 T cells in (**A**–**C**) peripheral blood and (**D**–**F**) spleen samples from WT and CCR2RKO mice (*n* = 5/group). (**G** and **H**) Splenic CD4+Foxp3+ Treg populations were also comparable between WT and CCR2RKO mice (*n* = 3/group).

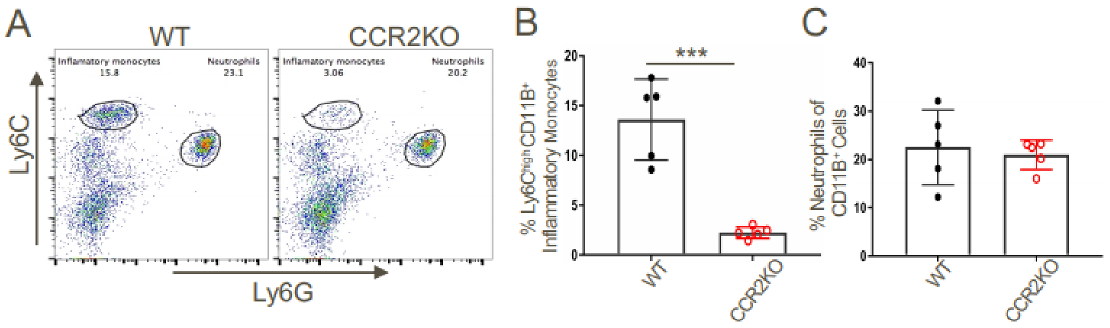


**Figure S3.** CCR2 gene deletion significantly reduced the frequency of splenic Ly6C$^{high}$ inflammatory monocytes. Evaluation of myeloid lineage cells in spleens from WT and CCR2KO mice revealed that (**A**,**B**) the frequency of splenic CD11b+ Ly6C$^{high}$ cells was significantly compromised in CCR2KO (*** $p < 0.001$), whereas (**C**) neutrophil populations were comparable (*n* = 5/group).

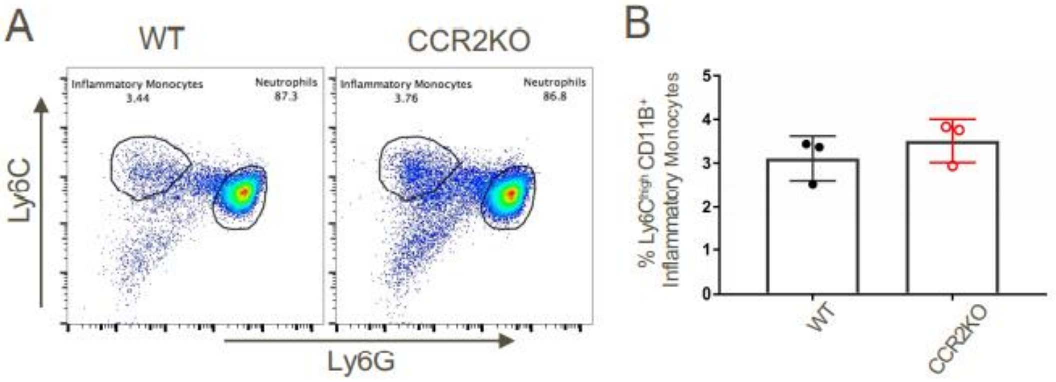


**Figure S4.** The frequency of CD11b+Ly6Chigh inflammatory monocytes was comparable in the bone marrows of WT and CCR2KO mice suggesting that the compromised frequency of these cells in the periphery was not attributable to a developmental defect in their production in CCR2KO mice.

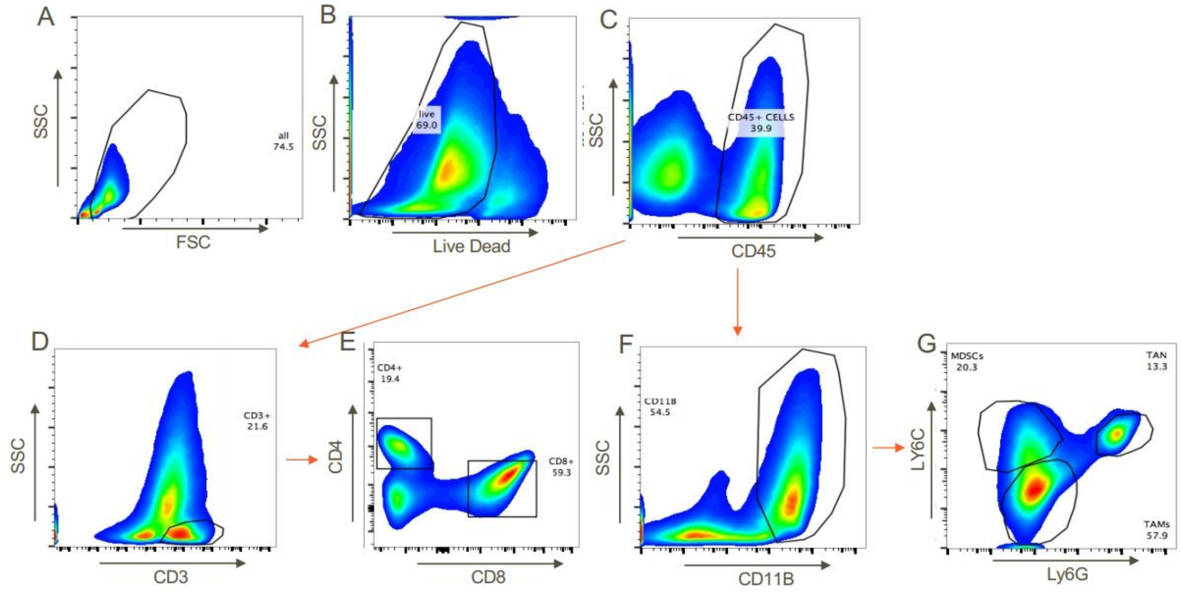


**Figure S5.** Gating strategy used to evaluate T cells and myeloid lineage populations in TC1 tumors.

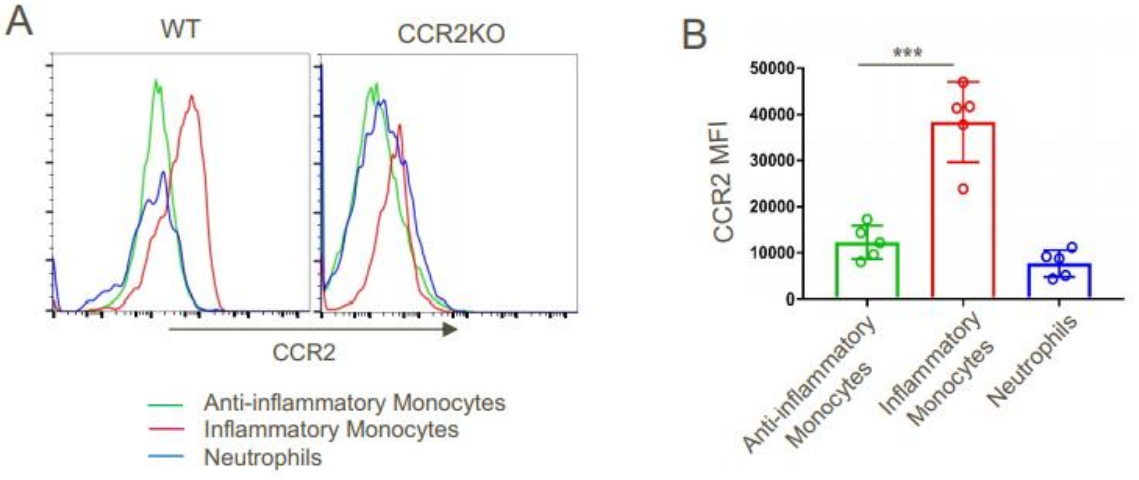


**Figure S6.** Blood CD11b+ Ly6Chigh inflammatory monocytes expressed higher levels of CCR2 than anti-inflammatory monocytes or neutrophils. Flow cytometry performed with peripheral blood samples from 5 mice/group (*** $p < 0.001$).

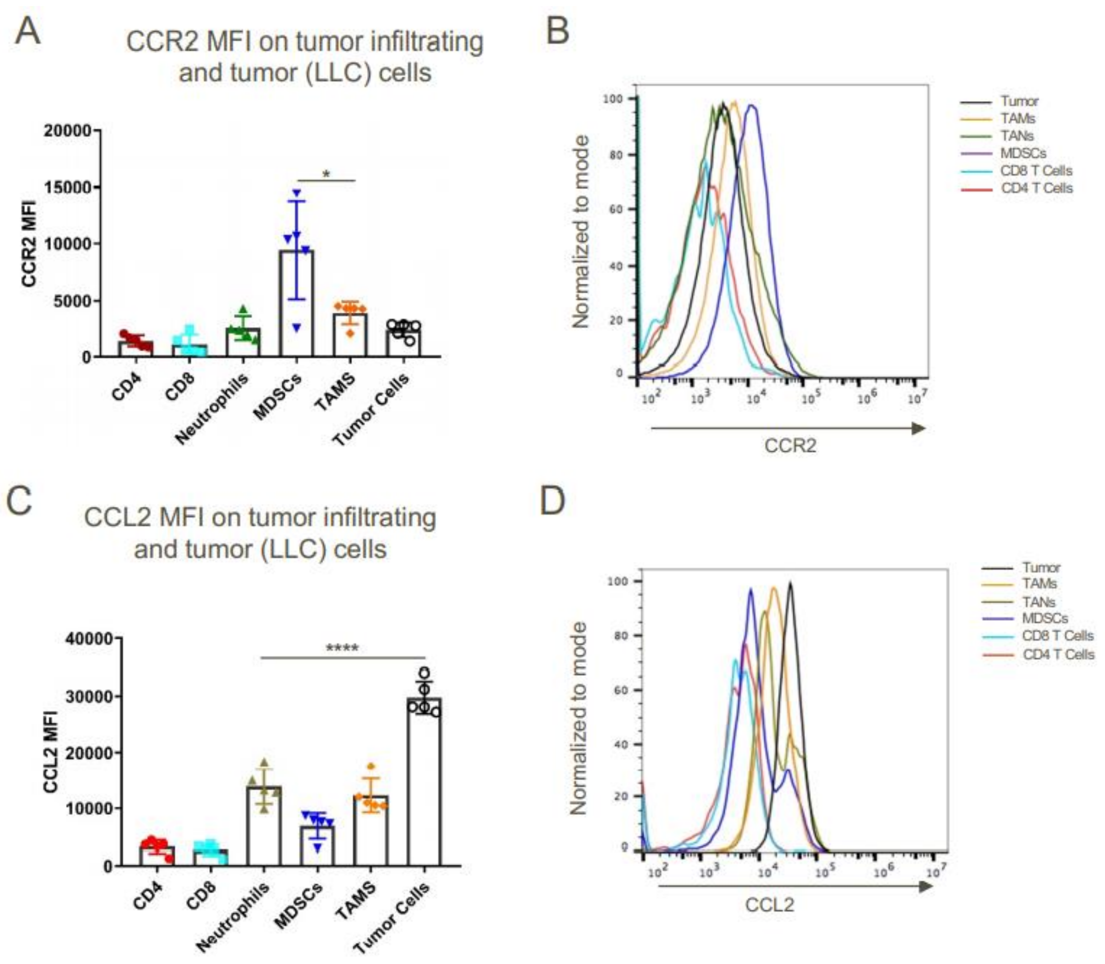


**Figure S7.** MDSCs associated with subcutaneously transplanted Lewis lung carcinoma cells expressed the highest levels of CCR2 whereas tumor cells were the main source of CCL2 (MCP-1).

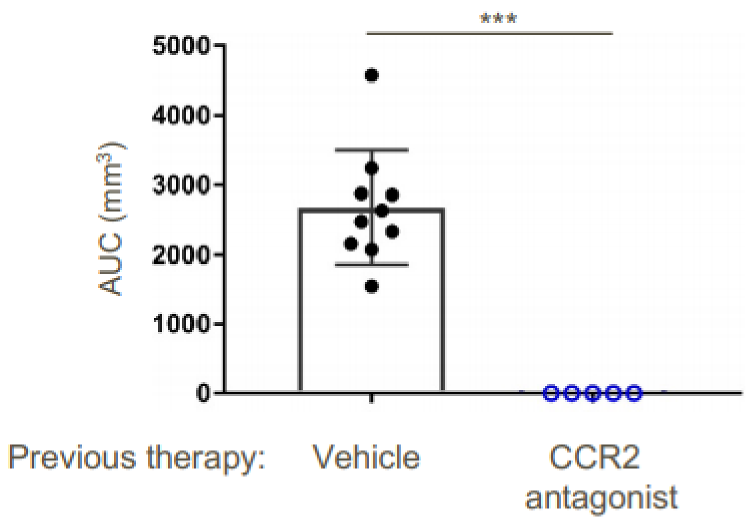


**Figure S8.** Mice that rejected the TC1 tumors in response to therapy with a CCR2 antagonist also developed memory responses. Control-treated mice and CCR2 antagonist-treated mice were re-challenged with fresh injection of TC1 tumor cells 30 days after cessation of the initial experiment. Tumor growth was monitored until day 13 and shown as AUC analysis (*** $p < 0.001$).
